# Perfluorooctanoic acid serum concentrations and half-lives in a community exposed to contaminated drinking water in New York State

**DOI:** 10.1038/s41370-025-00769-z

**Published:** 2025-04-17

**Authors:** Elizabeth L. Lewis-Michl, Steven P. Forand, Wan-Hsiang Hsu, Sanghamitra S. Savadatti, Ming Liu, June Moore, Qian Wu, Elizabeth J. Mullin, Kenneth M. Aldous

**Affiliations:** 1 New York State Department of Health, Bureau of Environmental & Occupational Epidemiology, Division of Environmental Health Assessment, Albany, NY USA; 2 University at Albany, Department of Epidemiology & Biostatistics, Rensselaer, NY USA; 3 New York State Department of Health, Wadsworth Center, Division of Environmental Health Science, Albany, NY USA

**Keywords:** Perfluorooctanoic acid (PFOA), Perfluorooctane sulfonate (PFOS), Drinking water contamination, Biomonitoring, Half-life

## Abstract

**Background:**

Investigations during 2014–2016 in two communities in New York State showed perfluorooctanoic acid (PFOA) in a public system serving 3800 residents (Hoosick Falls) averaging 534 ppt and in a smaller system serving 200 residents (Petersburgh) averaging 92.5 ppt. Bottled water (2015–2016) was provided until filtration brought PFOA levels to non-detectable (2016–2017).

**Objective:**

The New York State Department of Health (NYSDOH) sought to address community questions about exposures and evaluate reductions in serum concentrations.

**Methods:**

NYSDOH tested serum PFOA in 2016 just after drinking water exposure mitigation and again in 2018. Descriptive statistics for serum PFOA by sex, age, length of residence, and water consumption were evaluated using multiple regression, and half-lives were estimated.

**Results:**

Using the serum PFOA GM and median for tests occurring within 3 months of exposure mitigation (*N *= 1121) (47.5, 54.2) produced serum to water ratios of 89.0 and 101.6. A total of 1573 Hoosick Falls public water consumers (337 <age 18) participated in 2016 baseline testing. PFOA GM was 42.1 mcg/L (ppb) (CI: 39.9–44.4), 27 times higher than the U.S. population, from NHANES, 2015–2016. Concentrations were significantly higher for males, older ages, longer residence, and higher water consumption in multivariate regression. Among children, concentrations were highest among the youngest, <3 years. Half-lives for 307 participants tested in 2016 and 2018 produced a GM half-life of 2.86, adjusted to the Hoosick Falls age distribution. GM half-lives increased with age, from 1.96 years (CI: 1.69–2.27) for youth under 18, to 3.55 years (CI: 3.27–3.87) for people over 60.

**Impact:**

- This biomonitoring project assisted communities with PFOA-contaminated drinking water by providing comparative exposure information and tracking body burden reductions to confirm exposures were minimized.
- These data are also critical for filling gaps in knowledge about PFOA modes of action and for the conduct of studies that can identify exposure concentrations associated with health risks.
- The detailed PFOA serum findings described here are being used to construct and validate pharmacokinetic models that will estimate exposures over the lifespan.
- These findings provide a foundation for PFOA exposure assessment that will benefit the national Multi-Site PFAS Health Study and future studies as well.

## Introduction

Per- and polyfluoroalkyl substances (PFAS) comprise a large class of synthetic chemicals used extensively in commercial and industrial products. The two most widely used historically, perfluorooctanoic acid (PFOA) and perfluorooctane sulfonate (PFOS), are detectable in the blood of nearly the entire populations of developed countries due to their persistence in the environment and long biological half-lives [1]. Human and animal studies provide evidence of causal links between PFAS exposures and varied health effects, with large gaps remaining regarding modes of action and relevant exposure concentrations in humans [2–4]. PFAS’ relatively long biological half-lives are indicative of complex relationships among factors associated with their biological effects and elimination from the body [5]. The design of human exposure and health studies to fill gaps in our knowledge about health effects requires a more thorough understanding of factors affecting PFAS elimination rates and half-lives.

This report provides information about the sources of PFOA drinking water contamination in Hoosick Falls and Petersburgh, Rensselaer County, New York State (NYS), communities now included in the national PFAS Multi-Site Study (MSS), currently underway [6]. Serum PFOA concentrations and half-life estimates are presented for residents of the Village of Hoosick Falls served by drinking water with PFOA as the predominant contaminant. Detailed descriptive statistics are provided for two rounds of biomonitoring, in 2016 and 2018, before the national MSS commenced.

The New York State Department of Health (NYSDOH) conducted PFOA blood testing in these communities in response to strong community interest, to characterize exposures, and to potentially inform future health studies.

## Background

PFOA’s primary use was for manufacturing fluoropolymer products such as Teflon-coated fabrics and plastics. PFOS often predominates in fire-fighting foam but can also be the largest PFAS contributor at industrial sites. Exposures to PFAS can occur from industrial emissions to air, soil and water; from consumer products with oil-, stain-, and water-resistant properties, such as carpeting, furniture, clothing, food packaging, water repellant sprays; and personal care products [7]. The primary route of exposure is thought to be diet, particularly from water ingestion in areas with contaminated water [4].

PFAS are ubiquitous in the environment because of their widespread use and persistence. While PFAS concentrations are typically highest in environmental media and wildlife near manufacturing facilities and contaminated sites, emissions from such facilities often have significant impacts to groundwater and surface water over large geographic areas [7]. As concern about PFAS has grown, particularly since the early 2000’s, investigations launched near PFAS manufacturing or other industrial facilities (plastic and textile coating, metal plating, leather tanning), airports, fire-training centers, landfills, and wastewater treatment plants have identified a variety of types of PFAS emission sources and contaminated sites [8, 9].

PFAS concentrations in drinking water are strong predictors of PFAS serum levels across the spectrum of drinking water PFAS concentrations [10, 11]. The United States Environmental Protection Agency’s (USEPA) unregulated contaminant monitoring rule (UCMR) mandates public water system testing for specific contaminants in five-year cycles, and PFAS were included in the 3^rd^ UCMR, launched in 2013 (6 PFAS), and the 5^th^ UCMR launched in 2023 (29 PFAS) [12, 13]. Between UCMR 3 and UCMR 5, USEPA’s minimum reporting levels (MRLs) based on laboratory capability were reduced from 20 parts per trillion (ppt) for PFOA and 40 ppt for PFOS to 4 ppt for each. UCMR 3 showed 2.4% of public water systems detected PFOA above the MRL (20 ppt) and 1.9% detected PFOS above the MRL (40 ppt) [14]. UCMR 5’s preliminary summary data (based on 15% of U.S. public water systems) now show PFOA above the MRL (4 ppt) in 9.5% of public systems and PFOS above the MRL (4 ppt) in 10.7% of public systems [13].

Health Advisories and Enforceable Standards: When the drinking water system in Hoosick Falls was first tested for PFAS, the USEPA (non-enforceable) provisional short-term health advisory levels for PFOA and PFOS were 400 ppt and 200 ppt respectively [15]. In May 2016, USEPA issued Lifetime Drinking Water Health Advisories for PFOA and PFOS of 70 ppt separately or combined [16]. In June of 2022, USEPA issued interim Drinking Water Health Advisories of 0.004 ppt and 0.02 for PFOA and PFOS, respectively; in 2024, considering feasibility, USEPA promulgated maximum contaminant levels for PFOA and PFOS at 4 ppt each [17]. In 2020, New York State established enforceable PFOA and PFOS public drinking water standards of 10 ppt for each chemical [18].

Health Implications: Studies of PFAS in animals show hepatic, immune, reproductive, developmental, and carcinogenic effects [3, 19]. Impaired mammary gland development was observed in mice exposed to PFOA. Developmental effects of PFOA, PFOS and other types of PFAS exposures in animals include decreases in pup body weight and survival, and alterations in locomotor activity in rats and/or mice [3]. Human health studies associate PFAS exposures, including PFOA exposures, with increased cholesterol levels, lowered antibody response to vaccines, changes in liver enzymes, increased risk of high blood pressure or pre-eclampsia in pregnant women, small decreases in infant birth weights, and increased risk of kidney and testicular cancer [20, 21]. USEPA classifies PFOA and PFOS as “likely to be carcinogenic to humans” [22]. The International Agency for Research on Cancer has classified PFOA as carcinogenic to humans and PFOS as possibly carcinogenic to humans [23].

Half-lives: Half-lives in animals for PFOA and PFOS are generally much shorter than in humans, with PFOA half-life estimates for the rat, mouse, and monkey ranging from 2–4 hours to 30 days [2]. A recent systematic review of PFAS half-lives in humans included seven studies of workers and communities in a meta-analysis, and showed PFOA mean half-lives ranging from 1.48 to 5.1 years, mean of 2.75 years (CI: 1.88, 3.6) [24–31]. USEPA’s technical support document for the promulgation of a PFOA maximum contaminant level (MCL) utilizes a half life of 2.7 years based upon Li et al. [32] and a number of other considerations [22].

Community Studies: PFAS biomonitoring studies have been conducted in at least 15 communities with legacy PFAS in drinking water, most often PFOA, PFOS, perfluorohexane sulfonic acid (PFHxS), and perfluorononanoic acid (PFNA), as primary contaminants. These investigations, identified from peer-reviewed publications and government agency reports, include responses to varied contamination scenarios, from manufacturing facilities [33–39], fire-fighting foam [32, 40–44], and waste-water sludge [45, 46] (Supplementary Table 1).

A small number of these community studies conducted multiple blood testing rounds and evaluated PFAS half-lives. PFOA is among the PFAS evaluated for half-life estimation in the C-8 studies [25, 47], Veneto (Italy) [48], Ronneby (Sweden) [26, 32], Decatur (Alabama) [45], and Arnsberg (Germany) [49], with estimates of PFOA half-life ranging from 2.3 to 3.9 years. PFOA mean concentrations at baseline ranged from 16.3 ppb (Decatur) [45] to 180 ppb (C-8 follow-up including Little Hocking and Lubeck) [25] (Supplementary Table 2).

## Environmental contamination

Hoosick Falls: The Village of Hoosick Falls is in eastern Rensselaer County, New York (NY), approximately 35 miles northeast of Albany. The 2020 Census shows Village population of 3216, 97% identified as white. Median household income is $63,811, compared to the NYS median of $81,386 [50]. The Village is located within the Town of Hoosick, total population 6711, including the Village [50].

Sampling of the public water system by a local resident in 2014 and confirmatory sampling by the Village of Hoosick Falls and NYSDOH in 2014 and 2015 showed drinking water was contaminated with perfluorooctanoic acid (PFOA). The PFOA concentrations for 25 samples taken November 4, 2014 to February 18, 2016 ranged from 422 ppt to 983 ppt, averaging 533.6 ppt (Supplementary Table 3). A related chemical, perfluoroheptanoic acid (PFHpA), was detected in 19 of 25 samples, with highest concentration of 13 ppt and average of 10.4 ppt. In every sample from the public water system, PFOA comprised more than 95% of the total concentration of per- and polyfluoroalkyl substances (PFAS) tested. PFOA concentrations and trends over time prior to sampling in 2014 are not known, but the relatively small groundwater system appears to have provided homogeneous PFOA concentrations to households. PFOA concentrations were very similar among samples taken at varied locations in the distribution system prior to granular activated carbon (GAC) treatment in 2016 [51]. One central treatment facility received flow from a single well-mixed source at the wellfield first developed in the 1940s. Up to seven wells were used over the years, with the system generally served by two wells.

In late November 2015 free bottled water became available to residents, and on February 16, 2016, an interim GAC filtration system was fully operational. After a system-wide flushing program was completed, sampling showed no detections of PFAS in the water distribution system as of March 30, 2016. (The MRL at this time was 1.9–2.0 ppt.)

Local industrial manufacturing facilities, including Saint-Gobain Performance Plastics, which continues to operate in the village, used PFOA for many years. Products included circuit board laminates, polytetrafluoroethylene (PTFE)-coated fiberglass, and other PTFE products. Saint-Gobain is located near the Village wellfield and was identified as the source of PFOA contamination of the public water supply wells [52].

Petersburgh: The Town of Petersburgh, population 1372 in 2020, borders the Town of Hoosick, and is served primarily by private wells. The small hamlet of Petersburgh, located in the Town, has a public system serving approximately 200 residents (79 residences). Census data show the town’s population is 94% white with median household income of $77,639, compared to the NYS median of $81,386 [50].

NYSDOH collected samples from multiple public system supply wells on 2/13/2016. PFOA concentrations ranged from 39 to 120 ppt [53]. Free bottled water was provided to residents in February 2016 and continued until the GAC system was fully operational in January 2017. PFOA concentrations in drinking water system samples from 2016 and 2017, collected prior to GAC treatment, ranged from 84 to 106 ppt, averaging 92.5 ppt (Supplementary Table 3). Testing for additional PFAS has consistently shown the predominance of PFOA. Sampling of influent water prior to GAC treatment, in 2017, for example, showed PFOA comprised more than 95% of the PFAS tested, with PFOA at 106 ppt, PFHpA at 4.7 ppt, and non-detects for the other four PFAS tested. Taconic Plastics, which continues to operate in the town, was identified as the source of PFAS contamination [54].

Private Wells in Hoosick and Petersburgh: Private well testing was done by multiple labs, with PFAS MRLs ranging from 1.7 ppt to 2.2 ppt. PFOA was detected at reportable levels in 50% of the first approximately 1300 wells sampled in Hoosick and Petersburgh in 2015–2016. In the private wells with PFOA detections, 76% in Hoosick and 58% in Petersburgh showed concentrations below 70 ppt. In both towns combined, PFOA concentrations were higher than 600 ppt for about 3% of wells tested. Median private well PFOA concentrations were 25 ppt in the Hoosick area and 49 ppt in the Petersburgh area (Supplementary Table 3). The secondary contaminant was PFHpA, detectable in 21% of Hoosick wells and 19% of Petersburgh wells. PFOS was detected in 10% of wells in Hoosick and 2% of wells in Petersburgh. In Hoosick, when PFOS was detected, 89% of samples were below 10 ppt. PFOS concentrations varied more widely in the small number of Petersburgh wells with PFOS detections. When residents agreed to point of entry treatment, i.e., a GAC system on their private well, these systems were provided. Installations began immediately, along with regular testing and filter maintenance as needed.

Industrial Sources: The Saint-Gobain facility on McCaffrey Street in the Village of Hoosick Falls, operational since 1962 under several corporate owners, manufactured various polytetrafluoroethylene (PTFE) tape and fabric products variously labeled Teflon coated glass fabrics and fluoroglass. Until approximately 2003, the PTFE dispersions used at the facility contained PFOA. PTFE-coated fabrics were manufactured by dipping fabric into a liquid PTFE dispersion, then threading the coated material through rollers into heated towers. Up to eight oven-heated towers were used to heat the fabric at temperatures above PFOA’s boiling point, with gradual cooling as the fabric moved down the tower, a process known as sintering. Emissions to air from this process are thought to have contributed significantly to levels of PFOA found in soil and surface waters in the area. The disposal of the liquid dispersions and various cleaning and maintenance activities also contributed directly and indirectly to contamination of soil and groundwater on and near the site. Spent dispersions were disposed of into sewers at the site; some are alleged to have been shipped off-site for disposal [52].

The Taconic Plastics facility, located in the Town of Petersburgh, south of the Hamlet of Petersburgh, began operation in 1961. Like Saint Gobain, Taconic used PTFE dispersions to make Teflon-coated fabric. Taconic also used PFOA ammonium salt, surfactants, and other materials containing PFAS. The facility obtained a State Pollutant Discharge Elimination System Permit from 1989 to 2003 allowing discharges of process water containing residual PFOA and other PFAS. In 1997, Taconic began phasing out surfactants containing PFOA. Investigation of PFAS contamination at the site goes back to 2004 when Taconic initiated sampling for PFOA in groundwater at the facility [55].

## Methods

Initiation of blood testing: On February 13, 2016, free blood testing for PFOA began for current and former residents of the Hoosick and Petersburgh areas. For Hoosick area residents, blood testing began approximately 10 weeks after awareness of PFOA contamination in the public system became widespread and free bottled water became available. Blood testing began to be available to Petersburgh residents immediately after sample results showed PFOA contamination of private wells and the public supply. Approximately 2.5 years after baseline blood testing (February to November 2016), a second round was offered from June 2018 through March 2019. Five additional PFAS were tested in Round 2: PFOS, perfluorobutane sulfonic acid (PFBuS), PFHpA, PFHxS, and PFNA.

Eligibility and enrollment considerations: Individuals who currently or formerly lived or worked in the Hoosick and Petersburgh areas, whether served by public water or private wells, were eligible, with no restrictions on timing, length of potential exposures, or PFAS concentrations in private wells. For this report, findings for people with occupational exposures have been excluded. NYSDOH has previously provided limited summary information about PFOA concentrations for 169 participants who reported occupational exposures [38], and a separate report is being developed.

The NYSDOH Institutional Review Board (IRB) determined biomonitoring was being provided as a public health service and was officially exempt from IRB oversight. Study enrollment included an informed consent administered at blood collection events. This consent included options for storage of left-over samples for future use.

Sample collection: NYSDOH held 22 blood collection events in Hoosick Falls and two in Petersburgh from February 13, 2016 through Nov. 1, 2016. NYSDOH staff administered a brief survey that collected demographics, residential and occupational histories, water source and usage history, and information on prior and current health conditions. Supplies used to collect and process blood were tested prior to use to assure they were PFOA-free. Blood samples were drawn by trained nurses and phlebotomists (up to 20 mL for adults and 10 mL for children), allowed to clot for 45 min and then centrifuged to separate the serum. Serum samples were delivered to NYSDOH’s Wadsworth Center for analysis immediately following each event.

Laboratory analysis: Serum samples were received and accessioned by the Wadsworth Center’s Biggs Laboratory and stored at −30 °C until analysis. Human serum was analyzed using solid phase extraction followed by ultra-high-performance liquid chromatography - tandem mass spectrometry (UHPLC-MS/MS) according to the method, “Analysis of Perfluoroalkyl Substances in Human Serum by Solid Phase Extraction and Ultra High-Performance Liquid Chromatography Mass Spectrometry; LOAC-812SOP”. This CLEP and CLIA-approved clinical method is a modification of CDC Method 6304.04 (revised 11/03/13). Briefly, fifty microliters of serum are added into a Phree^TM^ 96-Well Plate containing isotopically labeled PFAS internal standards in acetonitrile. Sample matrix protein precipitation occurs followed by simultaneous extract filtration and phospholipid removal performed by a Perkin-Elmer Janus Liquid Handling System. After extraction, the sealed sample plates are placed in the UHPLC autosampler, and 10-µL portions of each extract are analyzed using an Agilent UHPLC-MS/MS (1290 UPLC with 6460C Triple Quadrupole Mass Spectrometer). PFAS compounds are separated on a Waters Acquity^TM^ UPLC BEH C18 column. For MS/MS, electrospray ionization in the negative-ion mode is used and selected reaction monitoring with specific precursor-ion and product-ion pairs for each analyte are used for detection of PFAS targets. Quantitation is achieved using the stable-isotope dilution technique with a series of ^13^C/^18^O/^2^H-labeled internal standards. A matrix-matched calibration curve is prepared in newborn calf serum over the range of 0.5–100 ng/mL. Samples with values that are above the top standard are diluted with newborn calf serum and re-extracted with the next sample batch.

Statistical Analysis: Serum PFOA measured at concentrations below the limit of detection (LOD) (LOD = 0.5 µg/L [ppb]) were replaced with ${LOD}/\surd 2$ following the National Health and Nutrition Examination Survey (NHANES) guidelines. Baseline geometric mean (GM) serum PFOA concentrations and their 95% confidence intervals (CIs), 50^th^ and 95^th^ percentiles for all participants, for adults 20 years and older, and for males and females separately, were computed for each drinking water source and provided along with 2015–16 NHANES concentrations [56]. Serum to water ratios for people served by public water at the time of blood collection were calculated using GM and median serum concentration for Hoosick Falls participants in the first three months of baseline sample collection and for the entire baseline group for Petersburgh due to small numbers.

Additional descriptive statistics are presented for participants served by Village of Hoosick Falls public water at the time of blood collection, including PFOA serum concentrations by sex-specific ten-year age groups, and PFOA concentrations for all adults by length of residence and water consumption. Multiple linear regression on the natural logarithm of PFOA concentration was used to evaluate associations between PFOA concentrations and age, sex, length of residence, and water consumption. Quadrant of residence within the water system was added to the model to evaluate the assumption of homogeneity of PFOA concentrations within the system.

Participants with serum PFOA concentrations below the NHANES 95^th^ percentile (4.17 ppb) at baseline were excluded from half-life estimation, thereby excluding any participants with apparent background exposure only. Half-lives were calculated using the equations below, where (k) is the first-order elimination rate constant, (Cp1) is the Round 1 PFOA serum concentration, (Cp2) is the Round 2 PFOA serum concentration, and (t2 - t1) is the time differential in fractional years between Round 2 and baseline sampling dates. (Additional analyses, currently underway, are addressing the issue of ongoing non-drinking water exposures’ effect on half-life estimates.)

$${T}_{1/2}=\frac{0.693}{k}$$

$$k=\frac{{{\rm{In}}}\left({\frac{{cp}1}{{cp}2}}\right)}{t2-t1}$$

For participants served by Hoosick Falls water at baseline who participated in Round 2, estimated half-life, half-lives by sex, and by age-group, are presented. An age-adjusted half-life for people served by Hoosick Falls water is estimated using the Hoosick Falls age distribution as the standard. All statistical analyses were performed with SAS software version 9.4.

## Results

From February 13 to November 1, 2016, 3411 people participated in baseline testing, and 1573 (48%) were currently served by Hoosick Falls public water, 51 (1.6%) by Petersburgh public water, and 1618 (50%) by private wells (currently or formerly) or by public water (formerly). The GM PFOA concentration for 1573 people served by the Hoosick Falls system is 42.1 ppb (CI: 39.9–44.4), 27 times higher than the US population GM of 1.56 ppb (CI: 1.47–1.66) [56] (Table 1). Aggregate results suggest females have lower concentrations than males, but the difference is not statistically significant. GM concentrations are statistically significantly higher for participants age 40–59 and older than 60 compared to younger age groups, and children under age 3 have a higher GM estimate than older children.

**Table 1:** Baseline serum PFOA concentrations (ppb) for Hoosick Falls participants, by sex and by age, and NHANES concentrations (2015–2016) for comparison.

	Hoosick Falls				NHANES		
		PFOA mcg/L (ppb)			PFOA mcg/L (ppb)		
	*N*	GM (95% CI)	P50	P95	*N*	P50	P95
All participants	1573	42.1 (39.9, 44.4)	47.0	195	1993	1.57	4.17
By sex							
Males	699	44.5 (41.1, 48.2)	50.5	205	964	1.87	4.07
Females	874	40.3 (37.5, 43.3)	44.8	188	1029	1.37	4.17
By age group							
<3	26	41.4 (27.6, 62.0)	41.6	^b^	^c^	^c^	^c^
3–5	52	27.2 (20.8, 35.5)	33.0	^b^	^d^181	^d^1.80	^d^5.58
6–11	120	26.4 (22.6, 30.8)	32.2	79	^d^458	^d^1.94	^d^3.84
12–19	165	25.5 (22.8, 28.5)	27.6	71	353	1.27	2.47
20–39	336	27.6 (24.7, 30.8)	30.9	150	549	1.47	3.57
40–59	481	52.3 (47.7, 57.3)	62.0	199	555	1.67	3.87
60+	393	70.1 (63.1, 78.0)	87.0	269	536	2.17	5.17

Source: NYSDOH Biomonitoring program.

*GM* Geometric mean, *CI* Confidence interval, *P50* 50^th^ percentile, *P95* 95^th^ percentile.

^a^Hoosick Falls samples collected February–November 2016.

^b^Hoosick Falls 95^th^ percentiles are not provided when the *N *< 120, to protect the confidentiality of individual results.

^c^ NHANES does not include children under 3 years in PFAS testing.

^d^ NHANES reports children’s results for 3–5 and 6–11 years only for 2013–2014 survey years.

The GM PFOA concentration for 51 people served by the Petersburgh system is 9.57 (CI: 7.33–12.5), 6.1 times higher than the US general population; and for the other group (*N *= 1618) comprising varied drinking water histories, the GM PFOA concentration is 7.16 (CI: 6.72–7.64), 4.6 times higher than the general US population (Supplementary Fig. 1, Supplementary Table 4). The GM serum PFOA concentration for participants served by Hoosick Falls public water is similar to that for Tuppers Plain, a C-8 Project community [33]; the GM for Petersburgh participants served by public water is similar to the GM for Bennington, Vermont [38] (Supplementary Fig. 2).

Serum to Water Ratios: Seventy-one percent of 1573 Hoosick Falls participants in baseline serum testing provided serum samples in the first three months of testing, February 13 through April 30, 2016. (Provision of free bottled water began in November 2015, and filtration of public system water began on February 16, 2016). The GM serum PFOA concentration for Hoosick Falls residents tested through April is 47.5 (*N *= 1121), 30.4 times the general US population concentration (1.56 CI: 1.47–1.66). Using this GM PFOA concentration (47.5) and an average PFOA concentration in drinking water of 533.6, the serum to water ratio for this group is 89.0. Using the median PFOA concentration (54.2) for participants through April produces a serum to water ratio of 101.6. For 51 Petersburgh public water system users, the GM of 9.57 and the average PFOA concentration in drinking water of 92.5 for the Petersburgh public system produces a serum to water ratio of 103.4. Using the median (11.3) increases the serum to water ratio to 122.2.

**Fig. 1:**
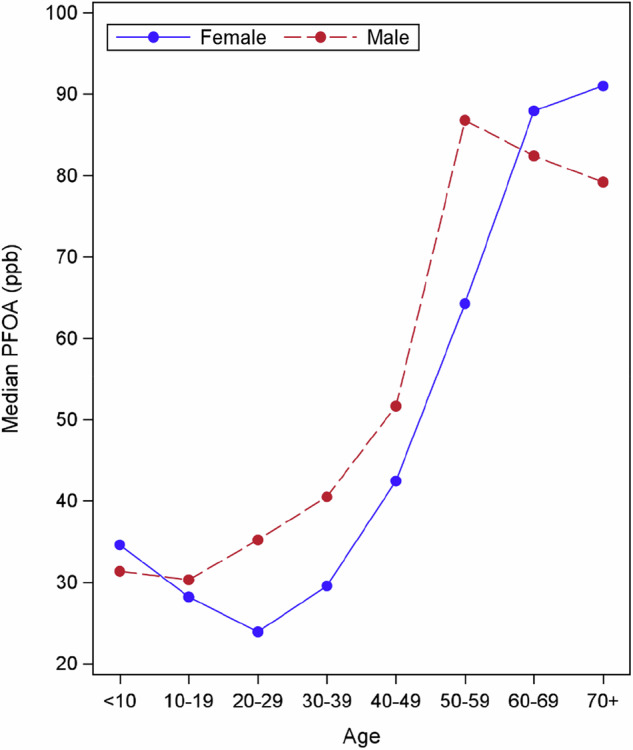
Median 2016 baseline serum PFOA concentrations for Hoosick Falls participants by age and sex (*N *= 1573). Source: NYSDOH Biomonitoring program.

Additional findings presented here focus on people served by the Hoosick Falls public system. Approximately 45% of Hoosick Falls residents participated in blood testing; the participants’ sex and age distribution is very similar to the Hoosick Falls distribution (Supplementary Table 5). 52% of participants had PFOA concentrations below 50 ppb and 95% had concentrations below 200 ppb (Supplementary Fig. 3). Table 2 shows how male versus female concentrations vary by ten-year age groups, with females age 20–29 having significantly lower concentrations than females 30–39 years. In this 20–29 year age group, males have significantly higher PFOA concentrations than females, 30.9 ppb (CI: 24.5–38.9) versus 18.6 ppb (CI: 14.8–23.3). Figure 1 illustrates a J-shaped trend, most pronounced for females, with relatively higher concentrations for the youngest group compared to mid-range ages, and the highest concentrations in the oldest age groups.

**Table 2:** Baseline serum PFOA concentrations for Hoosick Falls participants, by 10-year sex and age groups^a^.

	PFOA (ppb)		
	*N*	GM (95% CI)	P50
Males			
0–9	84	30.2 (25.2, 36.2)	31.4
10–19	90	26.4 (22.2, 31.3)	30.3
20–29	65	30.9 (24.5, 38.9)	35.2
30–39	79	35.3 (27.8, 44.7)	40.5
40–49	86	43.5 (34.2, 55.2)	51.7
50–59	115	72.8 (61.6, 86.1)	86.8
60–69	121	62.1 (51.2, 75.4)	82.4
70+	59	70.7 (51.3, 97.4)	79.2
Females			
0–9	74	26.6 (21.0, 33.6)	34.6
10–19	115	25.4 (22.4, 28.9)	28.2
20–29	78	18.6 (14.8, 23.3)	23.9
30–39	114	28.5 (23.5, 34.6)	29.6
40–49	100	36.8 (29.7, 45.4)	42.5
50–59	180	56.1 (48.8, 64.5)	64.3
60–69	128	75.5 (64.2, 88.8)	88.0
70+	85	74.2 (58.0, 94.8)	91.0

Source: NYSDOH Biomonitoring Program.

*GM* Geometric mean, *CI* Confidence interval, *P50* 50^th^ percentile, *P95* 95^th^ percentile.

^a^Hoosick Falls samples collected February–November 2016.

Table 3 shows serum PFOA concentrations for 1236 Hoosick Falls adults by length of residence and water consumption. PFOA serum concentrations generally increase with length of residence, but with an apparent plateau in the 20–39 year length of residence range. Serum concentrations for people consuming more than five 8-ounce cups (>1.2 L) of water per day were significantly higher than for those drinking fewer than 5 cups. While not statistically significant, results for the highest water consumption group, more than 10 cups (>2.4 L), suggest a decline or leveling, compared to PFOA concentrations for the middle group, 5–10 cups.

**Table 3:** Baseline serum PFOA concentrations for Hoosick Falls adults by length of residence and public water consumption.

	Number of participants	PFOA (ppb)		
		GM (95% CI)	P50	P95
All adults (age 18 and over)	1236	47.7 (44.8, 50.7)	57.3	208.0
Adults by length of residence on the Village water supply				
Less than 5 years	198	17.5 (14.9, 20.5)	17.9	111.0
5–9 years	169	42.0 (36.2, 48.7)	46.9	169.0
10–19 years	293	51.7 (46.4, 57.6)	61.1	196.0
20–29 years	222	63.4 (55.8, 72.0)	68.3	256.0
30–39 years	135	60.7 (51.1, 72.1)	74.3	207.0
40 years and more	219	75.6 (66.6, 85.8)	88.9	262.0
Water consumption per day^a^				
Less than 5 cups	366	36.1 (32.2, 40.3)	40.5	172.0
5–10 cups	477	59.4 (54.3, 64.9)	72.8	205.0
More than 10 cups	368	49.1 (43.6, 55.3)	58.2	263.0

The total does not equal 1,236 because of missing values.

*ppb* parts per billion, *GM* Geometric mean, *P50* 50^th^ percentile, *P95* 95^th^ percentile.

^a^Self-reported number of 8 ounce cups.

Table 4 presents the adjusted proportional changes for age, sex, years of residence and water consumption categories from multiple regression modeling of the natural logarithm of PFOA for adults on Hoosick Falls water. Consistent with the stratified findings, PFOA levels in adult participants were positively associated with older age, male sex, longer residence in Hoosick Falls, and increased water consumption. PFOA concentrations in males are significantly higher than in females and PFOA concentrations again appear to plateau for people with more than 20 years length of residence, and for people drinking more than 10 cups per day. We conducted an additional check on the assumption regarding homogeneous PFOA concentrations within the water distribution system. Quartile of residence was added to the multiple regression model, and was not significantly associated with serum PFOA concentrations (data not shown).

**Table 4:** Multiple regression analysis^a^ of participant age, sex, years of residence, and water consumption as predictors of baseline serum PFOA.

Variable	Beta	*p*-value	Adjusted proportional change (95% CI)
Age (years)			
18–39	reference		1.000 (reference)
40–59	0.438	<0.0001	1.550 (1.349, 1.782)
≥60	0.626	<0.0001	1.869 (1.596, 2.189)
Sex			
Female	reference		
Male	0.132	0.0193	1.141 (1.022, 1.275)
Years of residence on the Village of Hoosick Falls public water			
<10	reference		1.000 (reference)
10–19	0.594	<0.0001	1.811 (1.553, 2.112)
20–29	0.708	<0.0001	2.030 (1.717, 2.401)
30–39	0.693	<0.0001	2.000 (1.639, 2.439)
≥40	0.729	<0.0001	2.074 (1.726, 2.492)
Water consumption per day			
Less than 5 cups	reference		1.000 (reference)
5–10 cups	0.429	<0.0001	1.537 (1.345, 1.755)
More than 10 cups	0.371	<0.0001	1.449 (1.258, 1.670)

Hoosick Falls adults (*N* = 1236).

^a^From regression analyses on the natural logarithm of baseline serum PFOA concentrations.

2^nd^ Round Blood Testing: Of the 685 people who participated in Round 2, 316 were served by the Hoosick Falls water system when they participated in the first round. Round 2 blood collection occurred on average 2.5 years after baseline. Two of the additional PFAS not tested at baseline (PFBuS, PFHpA) had too few detections to calculate mean levels (Supplementary Table 6). The other three, PFHxS, PFNA and PFOS, thought to have longer half-lives than PFOA, were detected at levels almost identical to national population levels, confirming PFOA as the predominant contaminant, consistent with Hoosick Falls water samples.

For baseline to second round comparisons, we excluded 9 participants (2.8%) with PFOA concentrations below the 2015–16 NHANES 95^th^ percentile (4.17) at baseline [56]. In this group of 307 Hoosick Falls participants in both rounds, the baseline GM was 70.7 (CI: 63.8–78.4) and second round GM was 37.3 (CI: 33.2–42.0), a 42% reduction. The GM of 70.7 ppb for the group who participated in both Rounds (*N *= 307) is significantly higher than the geometric mean for the baseline group as a whole (*N *= 1573, GM 42.1, CI: 39.9–44.4) indicating participants with higher concentrations at baseline were more likely to participate in the second round. The age distribution was also different, with 47.9% of Round 2 participants age 60 or older compared to 25% at baseline and 22% in the Village population as a whole. The unadjusted estimated half-life for this group of 307, expressed as a GM, is 3.15 years (CI: 2.97–3.34), and as a mean, 3.65 years (CI: 3.34–3.95) (Table 5). See Supplementary Fig. 4 for the distribution of half-life estimates within this group.

**Table 5:** Serum PFOA half-lives for Hoosick Falls participants with repeat tests, by age group and sex (*N *= 307).

	Number of participants	Half-life (Years) P50	Half-life (Years) mean	Half-life (Years) GM
Age group				
0–17 years	29	1.95	2.10 (1.80, 2.39)	1.96 (1.69, 2.27)
18–39 years	38	2.47	3.44 (1.92, 4.96)	2.65 (2.17, 3.23)
40–59 years	93	3.19	3.59 (3.09, 4.09)	3.23 (2.96, 3.52)
60 and older	147	3.51	4.04 (3.67, 4.41)	3.55 (3.27, 3.87)
Sex				
Female	163	2.98	3.24 (2.99, 3.49)	2.90 (2.69, 3.12)
Male	144	3.28	4.10 (3.53, 4.68)	3.46 (3.16, 3.78)
Total	307	3.12	3.65 (3.34–3.95)	3.15 (2.97–3.34)

*GM* Geometric mean, *P50* 50^th^ percentile.

Half-lives for males are significantly longer than for females. Expressed as GMs, female half-life is 2.90 years (CI: 2.69–3.12) compared to male half-life of 3.46 years (CI: 3.16–3.78). Half-life estimates increase dramatically with age. The GM half-life for participants 0–17 years old (*N *= 29) is 1.96 (CI: 1.69–2.27) compared to GM half-life for participants 60 years and older (*N *= 147) of 3.55 (CI: 3.27–3.87). Direct age-adjustment of the half-life estimate, using the Hoosick Falls age group distribution as standard, produced GM and mean half-life estimates of 2.86 and 3.26 years respectively. (Additional analysis of these data is ongoing.)

For participants served by the Petersburgh public water system, which serves a relatively much smaller population than the Hoosick Falls system, fewer than ten participants were tested in both rounds, so additional analyses of this group are not presented here. An unadjusted 50^th^ percentile half-life for this group was estimated to be 3.2 years [57].

## Discussion

The serum test results show significantly elevated serum PFOA concentrations for participants served by Hoosick Falls and Petersburgh public water at the time of baseline and follow-up testing. People living in the surrounding area served by private wells, and people formerly living in the area, comprise a third group with more widely varying exposure scenarios, but on average, this group also showed significantly elevated serum PFOA concentrations.

Serum to water ratios: The serum to drinking water ratios estimated for Hoosick Falls (*n *= 1,121; ratio using serum GM: 89.0; using serum median: 101.6;) and Petersburgh (*n *= 51; using serum GM: 103.4, using serum median: 122.2) are similar to ratios estimated for Little Hocking public water users and C-8 private well users suggesting that people in these communities had exposure durations sufficient to achieve steady state levels [10, 58]. Emmett et al. published a serum to water ratio of 105 for Little Hocking water system users (*n *= 291), based on median PFOA serum concentration of 371 ppb and mean drinking water concentration of 3.55 ppb (3550 ppt) (14 water samples collected from 2002 to 2005) [10]. While there was awareness in the community about the contamination, free bottled water and/or GAC filtration had not yet occurred at the time these Little Hocking blood samples were collected [47]. Hoffman et al. estimated two serum to water ratios for a group from the C-8 Project communities served for 15 years or more by the same private wells (*N *= 108, 61 wells) using robust regression and steady-state pharmacokinetic modeling; regression produced a ratio of 141.5 versus pharmacokinetic modeling producing a ratio of 114 [58].

Holzer et al. reported PFOA contamination in drinking water in the Arnsberg Germany area similar to levels for Hoosick Falls, (500–640 ppt) when first sampled in 2006, yet serum PFOA levels there, tested in 2006, ranged from 22.1, 23.4, and 25.3 ppb for children, young women, and adult men, respectively [46], roughly half what might be expected compared to PFOA serum in the C-8 studies or this current study. Evaluation of stored serum from a German environmental specimen bank provides a plausible explanation, that Arnsberg area drinking water was contaminated for a relatively shorter time period, too short for concentrations to achieve steady state levels. PFOA concentrations in serum of 30 young adults, who were former Arnsberg area residents anytime between 1977 and 2004, showed no evidence of elevated PFOA exposure prior to 2005, suggesting PFOA exposures occurred for only two to three years before being discovered in 2006 [59].

Sex differences: Associations between Hoosick Falls residents’ PFOA concentrations and sex are consistent with most studies, showing higher concentrations in men than women, even when accounting for male occupational exposures. Sex differences are most apparent for the age-range of 12–50 years because of the role menstruation, cord-blood transfer during pregnancy, and breastfeeding play at those ages [60]. Studies suggest menstruation is the most important of these factors, but these factors alone do not appear to account for sex differences [61]. Ongoing research is exploring additional sex differences, including the role sex hormones play in renal transport processes, which are key for PFOA (and other PFAS) absorption and elimination [62–64].

Age differences: The J-shaped association between age and serum PFOA concentrations observed here is consistent with other community studies, including C8 communities [33]. PFOA is known to cross the placenta and has been shown to partition into breast milk [65–67], resulting in higher blood PFOA concentrations among the youngest age group, concentrations that tend to decline in early childhood due to rapid growth and other developmental changes [66, 68].

The non-linear association between PFOA concentration and age, and the different age patterns for females versus males are consistent with studies of the general population and communities with drinking water contamination [69, 70]. Higher concentrations among older age groups are likely to be partially due to the history of exposures over time, with older people having higher exposures prior to the legacy PFAS being phased out, beginning in about 2000. Of potentially greater importance for people with elevated exposures from drinking water is that age-related changes may slow the process of renal elimination. The glomerular filtration rate (GFR), a standard measure of kidney function, shows declining function (declining levels) with increasing age in the general population [71]. Having a normal vs non-normal estimated GFR (eGFR), was associated with shorter PFOA half-life in the Ronneby study [26]. Some studies show sex differences as well, with men having slightly higher (better) GFR levels that decline less rapidly with age compared to females [71]. This is a factor to explore regarding this study’s suggestive finding that PFOA concentrations for men begin to decline after age 60, while women’s concentrations continue to increase and surpass men’s concentrations after age 60 (Fig. 1).

A potential complexity that could interfere with the expected linear association between age-associated declining kidney function, reflected by eGFR, and higher serum PFOA concentrations is described by Jain and Ducatman [72]. Their study evaluated PFAS concentrations in the general population across categories of declining glomerular function, using NHANES data for 2007-2014 (*N *= 4057), and showed an inverted U-shaped distribution, meaning that at a more severe stage of kidney disease, declining kidney function was associated with lower PFOA concentrations, rather than higher concentrations [72] The analysis used four categories of estimated GFR, the first comprising normal kidney function and the next three comprising declining levels of eGFR, i.e., more severe kidney disease. Those with the most severe kidney disease, likely on dialysis, were excluded. For PFOA, the analysis showed higher PFOA concentrations for the 2nd and 3^rd^ categories of declining kidney function, compared to the first, i.e., normal kidney function; but significantly lower serum PFOA concentration for the fourth category, the most severe kidney disease group, compared to those with normal kidney function [70]. For males, PFOA serum concentrations began to decline at a less severe GFR stage, and declined more steeply, than for females [70]. Jain et al. caution that these non-linear associations between PFAS concentrations and kidney disease have implications for studies of PFAS concentrations and health outcomes that affect kidney function as well as studies of other clinical biomarkers affected by kidney disease [72].

The implication for this work is that individual PFOA serum concentration data collected at one time only, or at multiple time points but lacking information about concurrent kidney function, can be difficult to interpret from a cumulative exposure perspective as the biomonitored level may be elevated or lowered by the degree of kidney impairment. Further it points out that kidney function in addition to older age and other factors are likely important sources of variability in PFOA half-life estimates. Going forward, these repeat measures of serum PFOA will enhance the ability of the MSS to interpret exposure history and health effects for MSS participants.

Length of residence: The current study’s stratified results show markedly increased PFOA concentrations for adults with more than five years of residence in Hoosick Falls and suggest a leveling off after 20–29 years (Table 3). Some community studies show associations with length of residence as a proxy for exposure duration [40, 70]. Multiple regression controlling for age confirms these associations, showing an 80% increase in serum PFOA for people with 10–19 years length of residence compared to people with fewer than 10 years, and a leveling off after 20–29 years, or possible earlier. Rough estimation of the time to reach steady state as five times the half-life, using an estimate of 2.3–3.9 years from other community studies, suggests steady state would be reached on average between 12 and 20 years, consistent with this finding.

An important strength of this study is that PFOA concentrations in samples taken at varied locations within the Hoosick Falls distribution system prior to treatment as well a geographic analysis of serum PFOA concentrations confirm that PFOA exposures from Hoosick Falls public drinking water are relatively homogeneous throughout the system, supporting the use of length of residence as an exposure proxy. For most other community studies, drinking water concentrations are known to vary by location within the community, constraining the use and interpretation of length of residence for estimating exposures.

Water consumption rates: Drinking water concentrations and consumption rates have been reported to be the most important determinants of serum PFOA levels in communities with PFOA contamination in drinking water, but it is difficult to accurately ascertain water consumption using questionnaires. While not statistically significant, the serum data for Hoosick Falls suggest PFOA concentrations plateau for people drinking more than 10 eight-ounce cups (2.4 L) per day. The Pennsylvania community drinking water study suggests similar non-linearity, but the results were not statistically significant [40]. These non-significant but unexpected findings may reflect difficulties with participant water consumption reporting, an issue that warrants attention.

Half-life findings: This study’s estimation of PFOA half-life GM of 2.86, median of 2.77 years (using GM and median of individual half-life estimates, age-adjusted only) is close to estimates from other studies of communities with drinking water exposures. The C-8, Ronneby, and Veneto half-life studies used linear mixed effect models to account for sex, age, and other covariates, producing half-life estimates ranging from 2.3 to 2.99 years [25, 26, 32, 47, 48]. The shortest half-life estimate of 2.3 years is for the shortest follow-up time, one year, for a C-8 population that also had the highest PFOA concentration at baseline among these studies [25]. A second follow-up of an overlapping C-8 population, 3 to 4 years after baseline, produced a half-life estimate of 2.72 years [47]. Li, Andersson et al. evaluate half-life for Ronneby with up to ten samples per person over four years and show half-lives increasing over the four-year period, from 2.1 (1.78–2.67) for samples collected up to one year after exposure mitigation; to 2.39 (2.17–2.67), for samples collected 1–2.5 years after exposure mitigation; to 2.89 (2.57–3.3) for samples collected 2.5 years to 4.5 years after drinking water exposure mitigation, suggesting both time and dose-dependency [26]. The Veneto half-life estimate of 2.36 years is for a longer follow-up, and baseline at about four years post exposure, with the 2^nd^ sample approximately four years later. The Veneto participants are, however, markedly younger than either the C-8 or Ronneby participants, with age range from 14 to 52 years and median age, 28 years.

Brede’s half-life evaluation of the Arnsberg, Germany population calculates an unadjusted GM half-life of 3.26 years, quite similar to the other estimates despite an unusual sex-age distribution and some relatively low-level ongoing PFOA exposure from drinking water [49]. The Worley et al. study produced the longest half-life estimate among these studies, 3.9 years, and evaluated a longer timespan than most, six years. Worley et al. estimated PFOA half-life using a pharmacokinetic model that accounted for ongoing exposures, and they also suggest that PFAS exposures may have been declining for several years prior to the first blood collection [45].

All the published community studies that evaluated PFOA half-lives had approximately 50% female participants, but age distributions differed with some studies including children and the elderly, while others did not. Age adjustment can correct for some of the variation in age distributions in published PFOA half-lives, but the exclusion of children and people over age 60 has likely affected the estimates in some studies. The Bartell et al. study that produced the shortest half-life included more factors in its model than the other studies, factors such as use of bottled water and consumption of local produce, but it is not clear whether these modeling differences are related to the shorter half-life estimate [25].

Most of the community half-life studies referenced here do not adjust for ongoing (non-drinking water) exposures. An exception is the Ronneby study (PFOA baseline mean: 16 ppt) with adjusted half-life 17% shorter than non-adjusted half-life [26]. Addition of urine testing to serum biomonitoring allows direct measurement of PFAS clearance, thereby addressing unmonitored ongoing exposures [73]. Zhang et al. estimated a shorter half-life for PFOA (GM < 2) using this approach for a general population sample in China (*N *= 86) [74]. Together, these findings suggest the need for additional research addressing time, dose-dependency, and the role of ongoing exposures.

The current study observed longer half-lives for males and older age groups. Li, Andersson, et al. observed shorter half-lives for women under age 50 compared to men, and a fairly steady increase in half-life over the lifespan for both sexes [26], consistent with our findings. The recently published Veneto half-life study had a very large N, 5769 participants, and showed that male smokers and men in the highest category of alcohol use had longer half-lives, findings warranting follow-up.

General considerations: Important strengths of this PFOA serum testing study include the large number of participants, initial testing occurred close to the time when drinking water exposures were minimized, repeat sampling occurred approximately 2.5 years later, all samples were analyzed by the Wadsworth Center laboratory, and all age groups were included.

NYSDOH PFAS blood testing in Hoosick Falls and Petersburgh was a voluntary program, raising concerns about selection bias. The large proportional response (nearly 50% of Village population) provided a sample very similar demographically to the Village’s overall population, however. If selection bias played a role, it could be via an interaction with a confounding health problem like kidney disease: people with this condition could be more likely to participate and have higher PFOA concentrations and longer half-lives, depending on the distribution of kidney function within the group. The relatively large sample size in this investigation mitigates against this type of bias having a large effect, but the possibility of confounding remains.

This NYSDOH biomonitoring project did not collect data on the full range of factors that may affect PFOA concentrations, but did collect and evaluate the primary factors showing the strongest associations in other studies: sex, age, length of residence and water consumption. The half-life estimates presented here are descriptive and preliminary in nature, with more in-depth studies underway. The half-life estimates do not take account of potentially ongoing PFOA exposures. Given the overwhelming contribution of prior drinking water contamination compared to expected exposures from other sources, half-life estimates would likely not differ substantially, but the half-life estimates presented here may be slightly over-estimated [75].

The homogeneous distribution of PFOA throughout the Hoosick Falls public water system provides a foundation for exploring PFOA pharmacokinetics using these serum data, an effort currently underway. Variations in PFOA concentrations among Hoosick Falls residents can be assumed to be due primarily to timing and duration of Village residence in addition to amount of water consumed, age, sex, and related pharmacokinetics. This homogeneity of concentrations throughout the system is helpful for developing models for estimating drinking water exposures in years prior to any sampling for PFOA.

Baseline blood sampling for Hoosick and Petersburgh area residents occurred in 2016, with a second round in 2018, approximately five years and three years prior to the start of MSS enrollment. These data collected previously by NYSDOH provide a time series for serum concentrations prior to the measurement of serum PFAS, clinical biomarkers, and health status in the MSS. This is particularly important for PFOA and other legacy PFAS with long half-lives, complex renal processes, and potentially deleterious effects on the kidney. Individual PFAS serum concentrations are very useful as measures of exposure in PFAS health studies, but because serum concentrations can be influenced by factors other than exposure, time series data are critical [76, 77]. The limitations of personal biomonitoring can be addressed by using exposure measures that estimate an internal dose from environmental data or pharmacokinetic modeling, as was shown by Verner et al., for example [78], and the serum PFOA data collected by NYSDOH can inform the development of such models.

## Conclusion

The PFOA serum data collected in these two communities are a source of information about specific concentrations in individuals with varying characteristics and exposure histories, and can be used to construct and validate pharmacokinetic models that assist with estimating past exposures and PFOA toxicokinetics over the lifespan. PFOA serum testing was offered to these NYS communities primarily to inform individuals about their exposures, but these data can also contribute to understanding factors related to PFOA concentrations and human health effects. The PFOA serum concentrations showing expected reductions and the estimated half-lives confirm that actions taken to minimize PFOA concentrations in drinking water were effective for these communities. Ongoing work with these data will assist with the design and interpretation of health studies, including the MSS, currently underway, and thereby contribute to filling gaps in knowledge about PFOA exposures and health effects.

## Supplementary information

Supplementary table

Supplementary Figures

## Data Availability

The authors cannot make the data publicly available in summary or de-identified form due to biomonitoring data sensitivity and the danger of breaching confidentiality when small numbers are presented for subsets in follow-up research. Participants in the national CDC Multi-Site PFAS study who also participated in this study’s biomonitoring had the option to provide consent for their data to be used in the national study, so data are being made available as appropriate for that study.
