# Supplementary material for: Perfluorooctanoic acid serum concentrations and half-lives in a community exposed to contaminated drinking water in New York State
Source: J Expo Sci Environ Epidemiol. 2025 Apr 17;35(3):403–13. doi: 10.1038/s41370-025-00769-z (PMC12069094; doi:10.1038/s41370-025-00769-z)
Supplement: Supplementary file 1 — Supplementary table [file 41370_2025_769_MOESM1_ESM.pdf]

Supplement Table 1: List of sites identified in peer-reviewed publications and government agency reports with known PFAS contamination and community biomonitoring[a]

	Primary contaminant	Approximate date contamination discovered
Manufacturing		
C-8 Health Project, Mid-Ohio River Valley, Ohio & West Virginia (33)	PFOA	1984-2004
Washington County, Minnesota (34)	PFOS	2004
Paulsboro, New Jersey (35)	PFNA	2009
Hoosick Falls and Town of Hoosick, New York (36)	PFOA	2014-2015
Petersburgh, New York (37)	PFOA	2016
Bennington & North Bennington, Vermont[b] (38)	PFOA	2016
Belmont/Rockford & Parchment/Cooper, Michigan[b] (39)	PFOS	2017-2018
Fire-fighting foam		
Warminster & Horsham Townships, Pennsylvania (40)	PFOS	2013
Ronneby, Sweden (32)	PFOS	2013
El Paso County, Colorado (41)	PFHxS	2013-2016
Pease Air Force Base, New Hampshire (42)	PFOS	May 2014
Gustavus, Alaska (43)	PFHxS	2018
Newburgh, New York[b] (44)	PFOS	2016
Wastewater sludge		
Decatur, Alabama (45)	PFOS	2007
Arnsberg, Germany (46)	PFOA	2013

Notes:

[a]This list is likely not exhaustive; it includes sites identified in PubMed searches through 2023 using the terms biomonitoring, PFAS, PFOS, and PFOA.

[b]Government agency report. Government agency reports are not easily identified; the list here includes those known to the authors.

Supplement Table 2: Published community studies with PFOA half-life estimates: sample characteristics, methods and half-life estimates

Authors	Community & sample characteristics	End of exposure versus baseline	Repeat blood collection timing	PFOA mean at baseline (ppb)	PFOA 1/2-life mean (95%CI)	Adjustment:
Bartell et al. (ref 25)	C-8: 75% Lubeck, 25% Little Hocking<br>N = 172; Ages 18-89+<br>Baseline: June 2007	Lubeck: just after baseline; Little Hocking: 5 months after baseline	6 samples through August 2008, 1 year after baseline	180	2.3 (2.1-2.4) years	Timing of water filtration, water district, public, bottled water, sex, age, local and homegrown produce, public water at work
Li, Mucs et al. Appendix (ref 47)	C-8: Belpre, Tuppers Plain, Lubeck<br>N = 437<br>Age 20-60<br>Baseline: 2005-2006	Exposures end after baseline:<br>Belpre: Mar 2006<br>Tuppers Plain: May 2006<br>Lubeck: June 2007	2-3 samples, at 4.4 and 4.8 years after baseline, through Oct 2010	49.1	2.72 (2.52-2.90) years	Age, gender, BMI; date exposure ended applied to baseline blood sample, unless blood sample came after. time between water filtration start date and last blood sample was 3.9 years
Li, Fletcher et al. (ref 32)	Ronneby<br>N =106<br>Age 4 – 83<br>Baseline: June 2014	6 months before baseline	7 samples over 2 years, through Sept 2016	21.2	2.7 (2.5-2.9) years	Age, gender, BMI
Li, Andersson (ref 26)	Ronneby;<br>N = 114<br>Age: 4 - 84<br>Baseline: June 2014	6 months before baseline	Up to 10 additional samples over 4 years through May 2018	16	2.47 (2.27-2.70) years*<br>2.99 (2.79-3.21) years	Age, gender, BMI, background exposure*, time between exposure ending and blood sample collection
Brede et al. (ref 49)	Arnsberg<br>N = 65; 20 children 5-6;<br>22 mothers 25-47;<br>23 men 30-69<br>Baseline: Sept-Oct 2006	July 2006; 2 months before baseline	2 years after 1st sample, Oct-Nov, 2008	Children:22.4<br>Females 25.1<br>Males: 32.8	3.26 years GM)	(Unadjusted half-life); breakthrough creates some ongoing exposure
Worley et al. (ref 45)	Decatur<br>N=45<br>Baseline 2010<br>12 years+<br>Mean age: 56.6	Exposure ongoing<br>May have been declining before 2010	2nd samples 6 years after baseline, in 2016	16.3	3.9 years	Continuing PFOA intake; volume of distribution.
Batzella et al. (ref 48)	Veneto<br>N=5,769<br>Baseline 2017<br>Age 14-52<br>Median age 28	Aug 2013, about 4 years before baseline	2nd samples about 4 years after baseline, 2020-22	49	2.36<br>(2.33-2.40) years	Age, sex, BMI, smoking, alcohol, eGFR, education; (background PFOA level subtracted)

Supplement Table 3: Summary statistics for PFOA Concentrations in Hoosick and Petersburgh Area Public and Private Water Prior to GAC Treatment

	Number of People Served	Sampling Time Frame[a]	Number of Samples	Percent with PFOA Detected	Average or Median PFOA Concen-tration (ppt)[b]	Minimum Concen-Tration (ppt)	Maximum Concen-Tration (ppt)
Hoosick Falls Public System	3,216	11/4/2014 - 2/18/2016	25	100%	533.6	422	984
Petersburgh Public System	200	2/13/2016 - 9/28/2017	6	100%	92.5	84	106
Hoosick Area Private Wells	NA	2015-2016	1,019	50%	25.0	ND	>2,000
Petersburgh Area Private Wells	NA	2015-2016	314	50%	49.0	ND	>2,000

PPT = parts per trillion

Notes:

a. Sampling was either in a time period before GAC had been installed; or, if after GAC installation, sampling was from a location in the water system where water had not received treatment.
b. Among detections.

NA – Not Applicable. Private wells generally serve one household, but household size for each private well was not collected or estimated.

Source: NYSDOH

Supplement Table 4. Baseline serum PFOA concentrations for Hoosick and Petersburgh area participants by water source (samples collected Feb -Nov 2016), and NHANES general population concentrations (2015-2016)

	Hoosick Falls				Petersburgh				Other				NHANES		
	N	GM (95% CI)	P50	P95	N	GM (95% CI)	P50	P95	N	GM (95% CI)	P50	P95	GM	P50	P95
All participants	1573	42.1 (39.9, 44.4)	47.0	195.0	51	9.57 (7.33, 12.5)	11.3	[a]	1618	7.16 (6.72, 7.64)	6.58	75.0	1.56	1.57	4.17
Adults over 20	1210	48.1 (45.2, 51.2)	57.9	211.0	46	10.3 (7.70, 13.6)	12.7	[a]	1365	7.30 (6.79, 7.85)	6.61	79.2	1.60	1.67	4.27
Participants by gender															
Males	699	44.5 (41.1, 8.2)	50.5	205.0	25	13.2 (9.28, 18.8)	14.9	[a]	738	8.14 (7.39, 8.96)	7.51	86.7	1.80	1.87	4.07
Females	874	40.3 (37.5, 43.3)	44.8	188.0	26	7.02 (4.80, 10.3)	8.48	[a]	880	6.44 (5.90, 7.01)	5.77	59.1	1.36	1.37	4.17

[a]95th percentiles are not provided due to small numbers, to protect confidential participant results.
Sources: NYSDOH Biomonitoring program; NHANES[56]

Supplement Table 5. Comparison of Village of Hoosick Falls study participant demographics and overall Village demographics (2010 U.S. Census)

	Study Participants	Village of Hoosick Falls
Number of Residents	1,573	3,501
By gender		
Males	44%	47%
Females	56%	53%
By age		
Less than 3 years	2%	4%
3-5 years	3%	3%
6-11 years	8%	8%
12-19 years	10%	11%
20-39 years	21%	23%
40-59 years	31%	28%
60 years and older	25%	22%

Supplement Table 6. Second round serum PFAS concentrations for Hoosick Falls participants (with repeat PFOA tests) (N=316), and, for comparison, the general U.S. population age 12 and up (NHANES)[56]

	Participants served by Hoosick Falls public water			U.S. population 2015-2016	
PFAS	% of samples with PFAS detected	GM (ppb)	P95 (ppb)	GM (ppb)	P95 (ppb)
PFBuS	0%	NA	NA	NA	NA
PFHpA	0.3%	NA	NA	NA	0.20
PFHxS	88%	1.1	3.01	1.18	4.90
PFNA	60%	0.6	1.4	0.577	1.90
PFOS	98%	4.7	15.2	4.72	18.3
PFOA	99%	35.0	159.0	1.56	4.17

Abbreviation: NA= Not applicable; GM=Geometric mean; P95=95th percentile
Source: NYSDOH Biomonitoring program.

Supplement Figure 1. Serum PFOA concentration distributions at 2016 baseline for participants served by the Hoosick Falls public water, Petersburgh public water, or by private wells and/or previously by public water

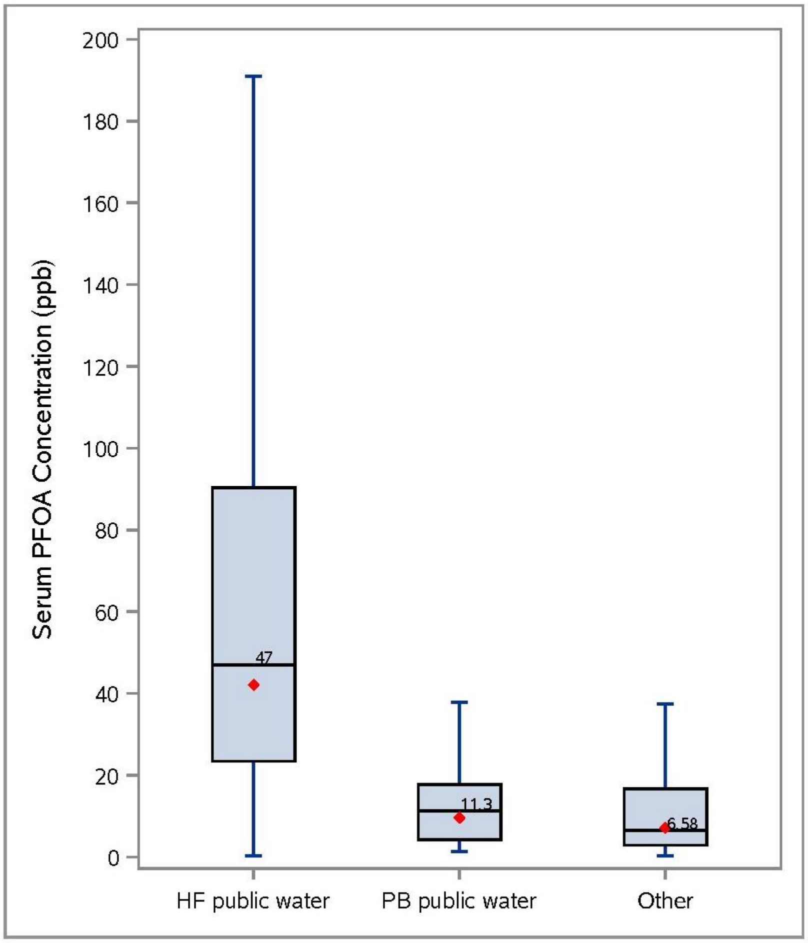

Hoosick Falls (HF) public water: n=1,573;
Petersburgh (PB) public water: n=51;
Private well/other: n=1,618.
Box plots indicate the geometric means (red diamond), medians (value provided) and interquartile ranges.
Source: NYSDOH biomonitoring program.

Supplement Figure 2. **Serum PFOA concentrations (geometric means) in ppb for selected U.S. communities with contaminated drinking water, and the U.S. general population**

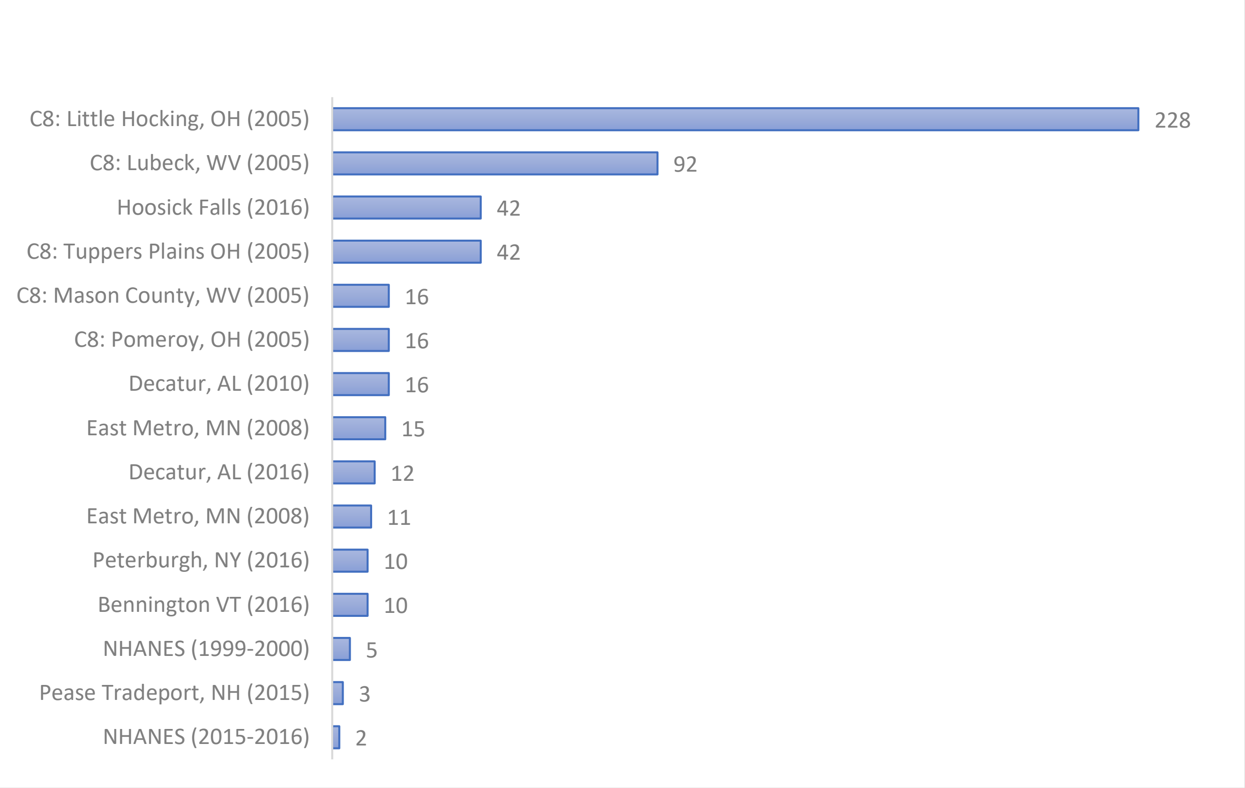

Sources:
OH and WV[33]; NH[42]; AL[45]; MN[34]; VT[38]; NHANES[56].
NYS Biomonitoring program.

Supplement Figure 3. Distribution of 2016 baseline serum PFOA concentrations among Hoosick Falls participants

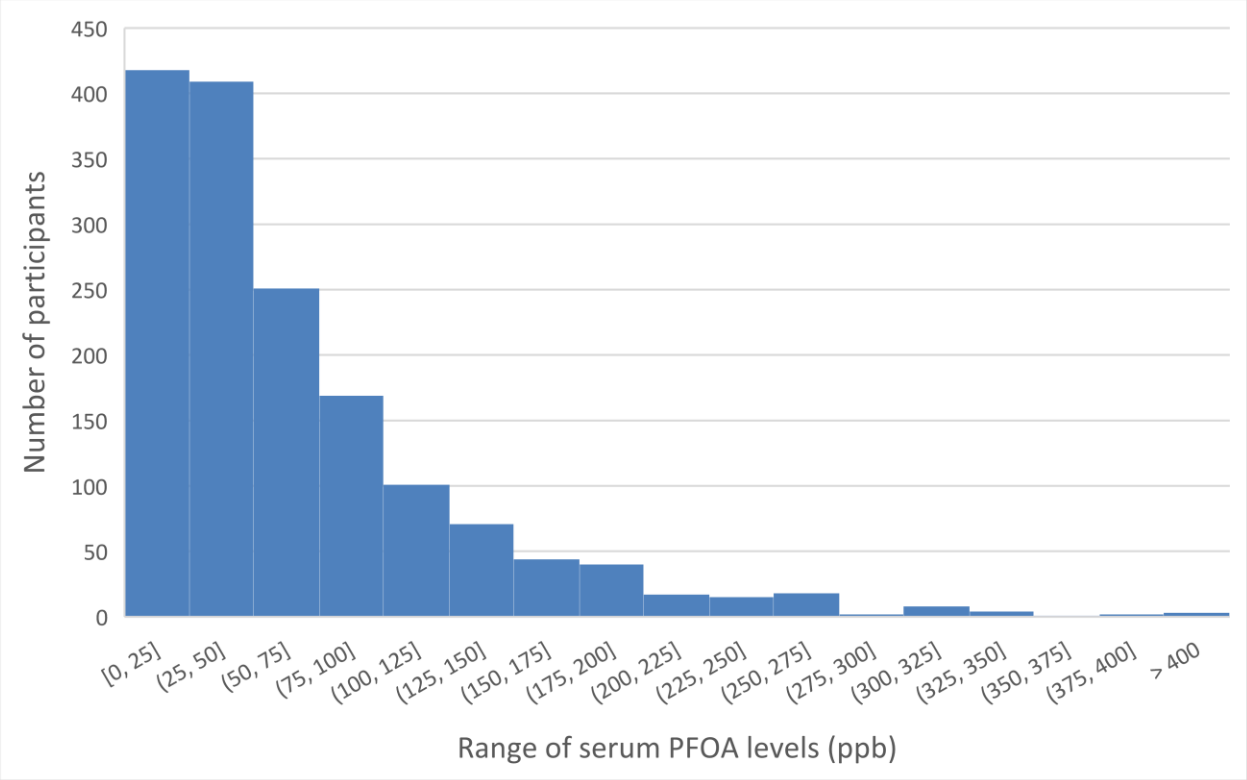

N = 1,573
Source: NYSDOH Biomonitoring program.

Supplement Figure 4: Serum PFOA half-life estimates for Hoosick Falls participants

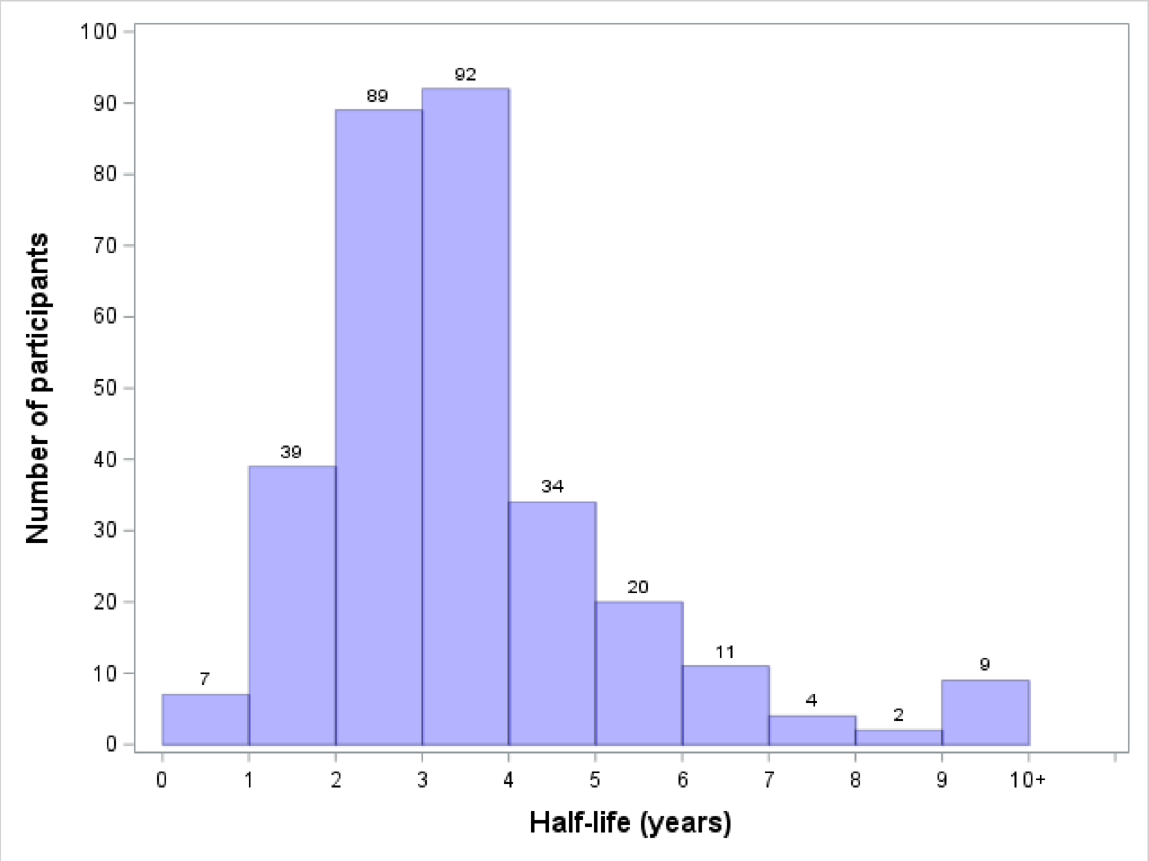

N=307
Source: NYSDOH Biomonitoring program.
