# Supplementary material for: Perfluorooctanoic acid serum concentrations and half-lives in a community exposed to contaminated drinking water in New York State
Source: J Expo Sci Environ Epidemiol. 2025 Apr 17;35(3):403–13. doi: 10.1038/s41370-025-00769-z (PMC12069094; doi:10.1038/s41370-025-00769-z)
Supplement: Supplementary file 2 — Supplementary Figures [file 41370_2025_769_MOESM2_ESM.docx]

**Supplemental Tables and Figures:** The supplemental table and figures are provided in one pdf file and include 6 tables and 4 figures that provide additional details about the biomonitoring studies reviewed, PFOA concentrations in drinking water sources, study participant demographics, PFAS serum test results, and PFOA half-life estimates.

**Supplement Table 1.** List of sites identified in peer-reviewed publications and government agency reports with known PFAS contamination and community biomonitoring

**Supplement Table 2.** Published community studies with PFOA half-life estimates: sample characteristics, methods and half-life estimates

**Supplement Table 3.** Summary statistics for PFOA concentrations in Hoosick and Petersburgh area public and private water prior to GAC treatment

**Supplement Table 4.** Baseline serum PFOA concentrations in ppb for Hoosick and Petersburgh area participants, by water source (samples collected Feb-Nov 2016), and NHANES general population concentrations (2015-2016)

**Supplement Table 5.** Comparison of Village of Hoosick Falls study participant demographics and overall Village demographics (2010 U.S. Census)

**Supplement Table 6.** Second round serum PFAS concentrations for Hoosick Falls participants (with repeat PFOA tests) (N=316) and the general U.S. population age 12 and up (NHANES)^56^

**Supplement Figure 1.** Serum PFOA concentration distributions at 2016 baseline for participants served by Hoosick Falls public water, Petersburgh public water, or by private wells and/or previously by public water

**Supplement Figure 2**. Serum PFOA concentrations (geometric means) in ppb for selected U.S. communities with contaminated drinking water, and the U.S. general population

**Supplement Figure 3**. Distribution of 2016 baseline serum PFOA concentrations among Hoosick Falls participants (N=1,573)

**Supplement Figure 4.** Serum PFOA half-life estimates for Hoosick Falls participants, N=307
